# OCT Results in Myopia: Diagnostic Difficulties in Clinical Practice?

**DOI:** 10.3390/jcm11123430

**Published:** 2022-06-15

**Authors:** Murgova Snezhana, Balchev Georgi

**Affiliations:** Ophthalmology Department, Medical University Pleven, 5800 Pleven, Bulgaria; snejana_murgova@yahoo.com

**Keywords:** OCT, myopia, hyperopia, RNFL thickness

## Abstract

Background: Optical coherence tomography (OCT) is a modern, non-invasive technique for examining the posterior segment of the eye in vivo. The quality of images is crucial for the diagnostic process. Despite good image quality and high signal strength, we still obtain images with less relevant diagnostic data, especially in relation to RNFL and GCL thickness in myopic and hyperopic eyes. Aim: To evaluate the change of RNFL, GCL thickness and rim and disk areas in myopic eyes that underwent OCT examination before and after refractive correction with contact lenses or glasses. Method: A prospective cross-sectional pilot study included 43 eyes in 22 patients with myopia and hyperopia, with or without astigmatism. Patients were examined using OCT with and without contact lenses or glasses. Results: RNFL thickness, GCL thickness, rim area and disk area average and minimum values were significantly changed after correction with glasses or contact lenses. Conclusion: Myopic patients with greater than −2.50 D have to be examined using OCT with their contact lens or glasses corrections in the case of borderline data. Uncorrected myopic eyes show a thinner RNFL and GCL and smaller disk areas, which may mislead ophthalmologists.

## 1. Background

Optical coherence tomography (OCT) is a modern, non-invasive technique for examining the posterior segment of the eye in vivo. It is a critical part of the diagnosis and follow-up of patients with macular and glaucoma disorders.

The technology is based on precise measurement of Retinal Nerve Fibre Layer (RNFL) and Ganglion Cell Layer (GCL) thickness based on cross-sectional images obtained with optical, ultrasonic and interferometry technology (as with Spectral Domain machines). The quality of images is crucial for the diagnostic process, and it depends on factors such as pupil size, quality of tear film, the presence of corneal and lens opacities, floaters, motion artefacts, the epiretinal membrane (ERM) and refraction, among others [1,2]. 

The complex interaction of all factors affects the signal strength, which is the main indicator of image quality during the study [3,4,5]. Despite good image quality and high signal strength, we still obtain images with less relevant diagnostic data, especially in relation to RNFL and GCL thickness [6,7,8,9]. This issue is increasingly addressed in the literature, especially the role of axial length, refraction, sex, age and other factors [5]. Every clinical ophthalmologist has been faced with the dilemma of whether a patient who has undergone OCT has glaucoma or whether the red sections of the result sheet are due to his or her myopia. All OCT devices have the option to correct the refractive error, but nevertheless, this diagnostic dilemma remains. We searched the literature for suitable articles, but the number was small, so we conducted our own research.

## 2. Aim

The aim of this paper is to evaluate the change of the Retinal Nerve Fibre Layer (RNFL), Ganglion Cell Layer (GCL) thickness and rim and disk areas in myopic and hyperopic eyes that underwent OCT examination before and after refractive correction with contact lenses or glasses.

## 3. Methods

Our prospective cross-sectional pilot study included 43 eyes in 22 patients with myopia and hyperopia, with or without astigmatism. All patients were Caucasians who were screened between December 2020 and May 2021 at our University Hospital. All the patients underwent comprehensive ophthalmologic examinations. Biomicroscopy, tonometry, ophthalmoscopy and autorefractometry were performed and best corrected visual acuity (BCVA) was obtained. The patients had no significant cataract, corneal or vitreous opacities or any significant retinal disproportions or ERM.

The patients were divided into two groups—myopic and hyperopic; all the patients were phakic. All patients underwent 2 sets of OCT fundus examinations: first, without refractive correction, and then performed with contact lenses or glasses based on BCVA. We used soft contact lenses and glasses with an anti-reflex filter included. In all the cases of contact lens examination, the dioptre equivalent for BCVA with glasses was taken into account. Before every examination, the pupils were dilated, and artificial tears were used.

All tests were performed on a Zeiss Cirrus 5000 HD-OCT-Angio machine with software Version 11.0.0.29946. The results and conclusions were only valid for that preset. All tests performed without refractive correction exploited the machine’s possibilities for automated refractive correction. All tests performed with refractive correction employed the autofocus function only. Therefore, equality of the optical correction in both groups was ensured.

We scanned all the patients with pre-set “ONH and RNFL OU Analysis: Optic Disc Cube 200 × 200” and “Ganglion Cell OU Analysis: Macular Cube 512 × 128” program profiles of Zeiss Cirrus 5000 HD-OCT. Patients with a signal strength of more than 7/10 were included. Spherical equivalent (SE) was used in all statistics tests.

The collected data were analysed with IBM SPSS Statistics 26. The normal distribution of the variables in the groups was checked with the Kolmogorov–Smirnov and Shapiro–Wilk tests. A standard descriptive analysis was also performed. The paired-sample *t*-test was used to compare means. Values of *p* < 0.05 were considered significant for all tests. Bivariate correlative analyses were done, and Pearson’s correlations were calculated. Regression analyses of Retinal Nerve Fibre Layer (RNFL) thickness and symmetry, disk and rim area, and average/min Ganglion Cell Layer (GCL) were performed to build regression statistical models.

## 4. Results

Of the 22 patients and 43 eyes examined, 34 eyes (79.1%) were myopic and 9 eyes (20.9%) were hyperopic. The mean age of the myopic patients was 50.11 years (range 22–84), and the mean age of hyperopic patients was 54.89 years (range 45–72). Distribution by sex was as follows: 6 male eyes (14%) and 37 female (86%). The hyperopic patients were all women.

In the myopia group, the mean spherical equivalent was −5.34 D, the median was −5.50 D, and the mode was −6.00 D; SD −3.35 D (from −0.75 to −14.00 D, with 95% confidence interval for mean −6.6 to −4.2). The chart of myopia is slightly left-skewed.

In the hyperopic group, the mean spherical equivalent was +2.41 D, the median and the mode +2.00 D; SD 1.03 D (from +1.00 to +4.00 D, with 95% confidence interval for mean 1.62 to 3.21). The chart is slightly right-skewed to almost Gauss-Laplase.

We included six pairs (before and after correction) on which statistical analyses were performed: average GCL, minimal GCL, RNFL thickness, RNFL symmetry, rim area and disk area.

The paired-sample *t*-test, which employed the means, showed significant difference before and after correction for average GCL, minGCL, RNFL thickness and disk area. No such difference was found in terms of RNFL symmetry and rim area with that test. Data are shown in Table 1, indicating significance. 

Bivariate correlative analyses were performed on the same pairs. The results before and after correction were all highly significant. Data are shown in Table 2 (*p* < 0.05 marked with * asterisk).

Data for hyperopia were highly significant, but due to the small number of participants presented in the paper, we focused on myopic patients only.

ANOVA/ANCOVA revealed a significant difference as well. However, we found the correlative and the regressive analyses much more predictive and comprehensive.

Linear regression analysis was done to present regression models, using the difference between observed pairs (before and after correction). From a clinical point of view, the difference value proved to be much more predictive rather than both parameters separately.

**Figure 1:**
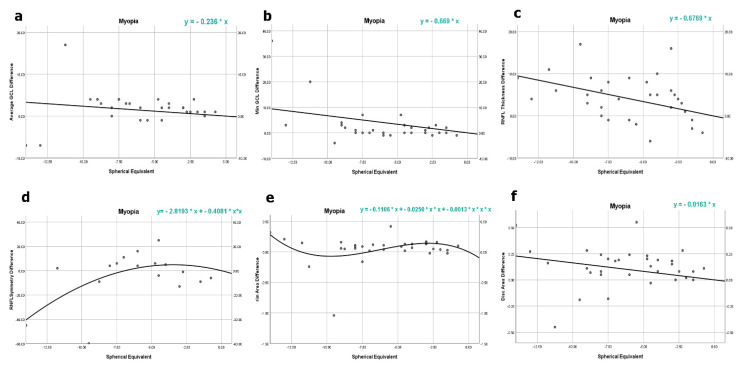
(**a**) Average GCL linear regression (*p* < 0.04); (**b**) min GCL linear regression (*p* < 0.001); (**c**) RNFL thickness linear regression (*p* < 0.001); (**d**) RNFL symmetry quadratic regression (*p* < 0.04); (**e**) rim area cubic regression (*p* < 0.05); (**f**) disk area linear regression (*p* < 0.002).

A set of regression analyses with scatter diagrams and equations are displayed in Figure 1, showing the results and the regression equations.

## 5. Discussion

OCT is a mainstay diagnostic method in clinical practice. The most common predictive parameters are related to the thickness of the RNFL and GCL layers and the areas of the rim and the disc. The myopic eye has a specific anatomy that complicates OCT examination and diagnosis establishment. 

**Figure 2:**
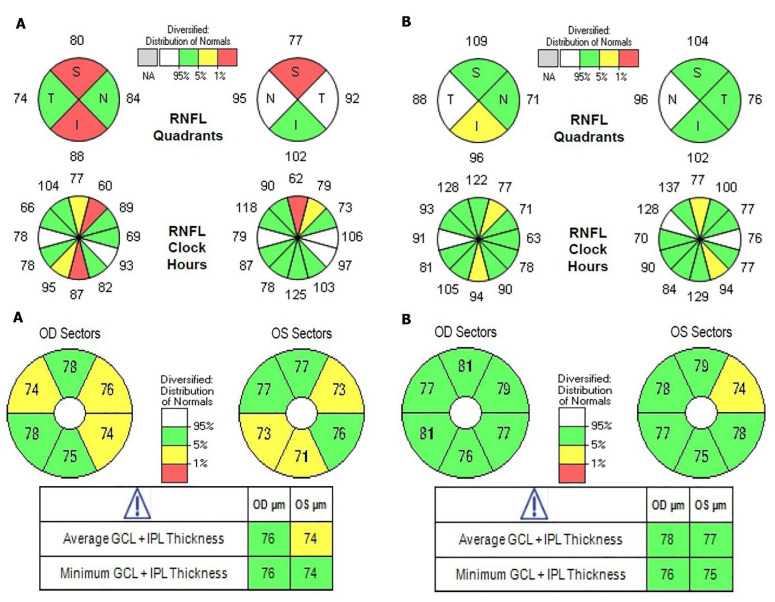
OCT scans of RNFL and GCL in a patient (**A**) before and (**B**) after correction.

We found that OCT performed on patients who have not undergone correction procedures may significantly change the clinical diagnosis [10,11]; a typical example is presented in Figure 2. A similar observation was reported by Berkenstock, who found that contact lenses improve the OCT scans [12].

On the other hand, it is known that the thickness of RNFL depends on the axial length of the eye. To avoid this effect, we statistically processed each eye separately. Thus, the only factor that changes in our study is the refraction; the axial length remains the same. To dispel doubts that dioptric correction, or lens material, may alter OCT readings, we relied on a study of healthy patients [13]. Other authors demonstrate a lack of thinning of RNFL after LASIK procedures on the cornea, as well as a slight or significant thickening, which supports our thesis [14].

We found statistically significant differences in the OCT scans of all the myopic eyes before and after correction in the six evaluated pairs for RNFL and GCL thickness as well as rim and disc areas. We observed thinner RNFL and GCL and smaller disc and rim areas in OCT scans before glasses or contact lens correction, even with the machine self-correction program applied. 

The hyperopia group showed significant differences for all parameters (before and after) as well, but these were not significant in the regression analysis, which is likely due to the small number of participants. Nevertheless, the association was evident in all tests. The hyperopic eyes showed an opposite direction compared to the myopic eyes. However, additional studies are required to prove such an assumption; we note the hyperopic data here with the aim to provoke research in that area. Astigmatism affects both myopic and hyperopic eyes, but it is likely to significantly affect hyperopic eyes.

We expected to build a regression equation that might be helpful in clinical practice. To support practice, we conducted the analysis not against the background of values after correction but against the difference between values before and after correction. All regression tests with difference values were significant, as presented in Table 3; the regressive analyses equations are shown in Figure 1.

The regression models that were developed were not all linear; some were cubic or quadratic. However, we obtained regression coefficients that helped us build every point in the regression line, presented on the scatter diagrams. The regression analysis shows that a dioptre greater than −2.50 D is a significant threshold for performing OCT with correction. Based on our equations, it is possible to calculate the relation between every single dioptre and changes in the thickness of the observed parameters.

## 6. Conclusions

We can conclude that all myopic patients with greater than −2.50 D have to be examined using OCT with their contact lenses or glasses corrections in the case of borderline data. The same tendency in the opposite direction was found regarding hyperopic patients, though further studies are required. OCT examination of uncorrected myopic eyes yields a thinner RNFL, a GCL difference and smaller disk areas; these are thicker on hyperopic eyes. A threshold of −2.50 D shows that accommodation may not play a significant role in OCT examination, as emmetropic and slightly myopic eyes did not produce significant data. Moreover, the regression equations obtained in that research article can be regressively used to calculate missing survey data from the very same or different cohort for certain diopter. For emmetropic and slightly myopic patients, who aren’t part of that survey, that can happen as well with the very same equations, and all that will show a significance of *p* < 0.005.

## Figures and Tables

**Table 1:** Descriptive analyses; PST: Paired-Sample *t*-test; N: number of cases; *p* < 0.05 marked with *.

	MYOPIA				HYPEROPIA			
Parameter	N	Without Correction Mean (SD)	With Correction Mean (SD)	PST	N	Without Correction Mean (SD)	With Correction Mean (SD)	PST
**Average GCL**	30	72.13 (8.94)	74.03 (7.67)	0.014 *	9	79.44 (7.384)	79 (7.297)	0.225
**Min GCL**	30	66.67 (16.21)	69.63 (12.95)	0.038 *	9	73.33 (16.371)	73.89 (14.03)	0.585
**RNFL thickness**	32	81.43 (9.476)	85.81 (9.29)	0.001 *	9	99.66 (11.87)	96.55 (9.63)	0.062
**RNFL symmetry**	17	61.47 (34.92)	57.47 (40.48)	0.438	4	77.75 (18.39)	81.5 (19.07)	0.172
**Rim Area**	34	1.15 (0.35)	1.20 (0.287)	0.158	9	1.38 (0.169)	1.3689 (0.16)	0.532
**Disc Area**	34	1.65 (0.46)	1.77 (0.44)	0.001 *	9	2.27 (0.27)	2.24 (0.30)	0.312

**Table 2:** Correlative analyses of the selected pairs before and after correction; N: number of cases; *p* < 0.05 marked with * asterisk.

	Correlations for MYOPIA before/after Correction					
	Aver GCL	Min GCL	RNFL Thick	RNFL Symmetry	Rim Area	Disc Area
**Pearson Correlation (R)** **(*p*-Sig.)**	0.897 * (0.001 *)	0.892 * (0.001 *)	0.840 * (0.001 *)	0.859 * (0.001 *)	0.775 * (0.001 *)	0.922 * (0.001 *)
**N**	34	34	32	17	34	34
	**Correlations for HYPEROPIA before/after correction**					
**Pearson Correlation (R)** **(*p*-Sig.)**	0.991 * (0.001 *)	0.993 * (0.001 *)	0.941 * (0.001 *)	0.976 * (0.024 *)	0.944 * (0.001 *)	0.966 * (0.001 *)
**N**	9	9	9	4	9	9

**Table 3:** Regression analyses comparing differences (before and after correction) with SE-Q = quadratic regression; C = cubic regression; ^a^ = significant for quadratic regression; ^b^ = significant for cubic regression; * = significant for linear regression.

	MYOPIA		HYPEROPIA	
**Linear Regression**	**R Square**	**ANOVA**	**R Square**	**ANOVA**
**Average GCL (Diff.)**	0.138	0.04 *	0.107	0.356
**Min GCL (Diff.)**	0.332	0.001 *	0.018	0.714
**RNFL thickness (Diff.)**	0.481	0.001 *	0.199	0.197
**RNFL symmetry (Diff)**	0.134 (Q = 0.348) ^a^	0.135 (Q = 0.04 *) ^a^	0.336	0.305
**Rim Area (Diff)**	0.027 (C = 0.213) ^b^	0.347 (C = 0.05 *) ^b^	0.077	0.439
**Disc Area (Diff)**	0.266	0.002 *	0.206	0.188

## Data Availability

Data are available on request due to privacy or other restrictions.
